# Sustainable Reduction of Sleepiness through Salutogenic Self-Care Procedure in Lunch Breaks: A Pilot Study

**DOI:** 10.1155/2013/387356

**Published:** 2013-12-05

**Authors:** Sebastian Schnieder, Sarah Stappert, Masaya Takahashi, Gregory L. Fricchione, Tobias Esch, Jarek Krajewski

**Affiliations:** ^1^Schumpeter School of Business and Economics, Experimental Industrial Psychology, University of Wuppertal, Gaussstraße 20, 42119 Wuppertal, Germany; ^2^National Institute of Occupational Safety and Health, Nagao 6-21-1, Tama-Ku, Kawasaki 214-8585, Japan; ^3^Harvard Medical School, Massachusetts General Hospital, Benson-Henry Institute for Mind Body Medicine, Warren 615, 55 Fruit Street, Boston, MA 02114, USA; ^4^Department of Medicine, Harvard Medical School, 25 Shattuck Street, Boston, MA 02115, USA; ^5^Division of Integrative Health Promotion, Coburg University of Applied Sciences, Friedrich-Streib-Straße 2, 96450 Coburg, Germany

## Abstract

The aim of the study was to elucidate the immediate, intermediate, and anticipatory sleepiness reducing effects of a salutogenic self-care procedure called progressive muscle relaxation (PMR), during lunch breaks. The second exploratory aim deals with determining the onset and long-term time course of sleepiness changes. In order to evaluate the intraday range and interday change of the proposed relaxation effects, 14 call center agents were assigned to either a daily 20-minute self-administered PMR or a small talk (ST) group during a period of seven months. Participants' levels of sleepiness were analyzed in a controlled trial using anticipatory, postlunchtime, and afternoon changes of sleepiness as indicated by continuously determined objective reaction time measures (16,464 measurements) and self-reports administered five times per day, once per month (490 measurements). Results indicate that, in comparison to ST, the PMR break (a) induces immediate, intermediate, and anticipatory reductions in sleepiness; (b) these significant effects remarkably show up after one month, and sleepiness continues to decrease for at least another five months. Although further research is required referring to the specific responsible mediating variables, our results suggest that relaxation based lunch breaks are both accepted by employees and provide a sustainable impact on sleepiness.

## 1. Introduction

Prevalence studies show that around 13% of the population suffers from increased sleepiness during the day [1]. Next to obesity and an impaired quality of life, in occupational settings, sleepiness also impairs motivation, mood, and job satisfaction and can lead to adverse health outcomes [2–7]. In occupational settings, self-care interventions are used to improve employees' well-being and health [8]. Four strategies comprise self-care programs: nutrition, behavior, exercise, and relaxation [9]. Relaxation activities mainly include relaxation response approaches [10] like, for instance, progressive muscle relaxation (PMR) [11–15]. While a substantial body of research has been conducted on recovery activities in assisted, controlled, and short-term laboratory contexts, to our knowledge, only few studies have addressed deactivating techniques on a self-administered (nonassisted), fully realistic long-term experimental level within the context of nonshift work and organizational daily life settings [16, 17]. Hence, the main aim of the present study is to analyze the recovery value of a promising but rarely evaluated salutogenic break-time activity: PMR. In detail, the study will determine whether PMR-based lunch breaks result in a sustainable, respectively, immediate, intermediate, and anticipatory effects of reduced sleepiness.

We expected PMR to reduce sleepiness-related symptoms, as PMR results in a general parasympathical mode of deactivation starting a wide variety of recovery processes by reduced cognitive, muscular, and cardiovascular activity [18–21]. Specifically, within laboratory settings, there are many well-documented recovery effects of relaxation and PMR on the neurobiological, cardiovascular, neuromuscular, electrodermal, autonomous, and central nervous processes. These, again, might lead to either a broadly energized state including reduced emotional, motivational, and cognitive strain or reduced physical and mental fatigue; together, they all might contribute to reduced sleepiness experiences [17, 22–24]. Due to both these theoretical and empirical hints connecting PMR with short-term mental and physical deactivation and its ability to counteract a broad range of cognitive, emotional, and motivational sleepiness-related symptoms [25], our preliminary hypothesis claims that *PMR-based lunch breaks result in immediate reduced sleepiness*.

Additionally, we suggest that due to necessary familiarization processes, referring to building trust in the relaxation setting and the nonnegative reactions of supervisors and colleagues, an onset of the sleepiness reducing effects might occur after several weeks of habituation [26]. Therefore, a further refined hypothesis suggests that *PMR-based lunch breaks result in immediate reduced sleepiness when familiarization has been completed.* As shown above, numerous studies have documented the recovery potential of systematic relaxation techniques in nonworksite research fields. However, these occupational self-care procedures [27] have never been implemented into organizational contexts before. After satisfying the needs of a realistic occupational implementation of PMR, our Hypothesis claims that *PMR-based lunch breaks result in immediate reduced sleepiness when familiarization has been completed and will then continuously increase for at least five more months.*

After proposing the expected long-term increase of the sleepiness effects over several months (“interday perspective”), it still remains unclear if the effects last several minutes (as demonstrated in several laboratory studies) [28] or if the impact of PMR endures for several hours (“intraday perspective”). Knowing the effects, half-life time helps to determine the total impact, total added value, and, thus, individual and organizational relevance, which are created by PMR breaks. In order to evaluate the total benefit, the analyzed effects have to be extended from immediate (several minutes) and intermediate (several hours) effects to anticipatory effects (next day). The anticipatory effect reflects a long-range impact of PMR on the sleepiness of the anticipatory status of the following day [17]. Our hypotheses can be summarized as follows: *PMR-based lunch breaks result in, respectively, immediate, intermediate, and anticipatory effects (intraday perspective) of reduced sleepiness when familiarization has been completed and will then continuously increase for at least five more months (interday perspective)*.

## 2. Method

### 2.1. Participants

All participants took part voluntarily. Participants consisted of inbound call center agents recruited in a medium-sized company in Germany dealing with queries about professional installations. Of the 30 agents working in the call center, 16 were chosen randomly, from which two declined to participate, resulting in a total sample of 14 participants (participation rate: 47%). Participants met the following inclusion criteria: (a) having worked as call center agents for more than six months, with a regular five-day schedule, 40 working hours weekly, and a fixed daily work schedule from 8:00 to 17:00; (b) being healthy and reported no sleep disturbances (e.g., due to having a newborn at home); and (c) having no prior experience in systematic deep relaxation procedures.

Through the use of randomization procedure, seven pairs, which had been matched in age (range ±4 years) and gender, were either assigned to the PMR or to the ST group. Participants in the ST group were told that they were put on a waiting list. Each group (ST and PMR) consisted of four male and three female participants. The mean age of the female participants was 35.0 years (SD = 9.5) in the PMR group and 34.5 years (SD = 7.0) in the ST group. The mean age of the male participants was 43.3 years (SD = 8.0) in the PMR group and 41.0 years (SD = 11.6) in the ST group.

#### 2.1.1. A Priori Group Equivalence Check

With regard to the relatively small sample, we tested the equivalence of the groups by calculating chi square tests (for sedative medication) and Mann-Whitney *U* tests for age, body-mass index (BMI), length of work within a company, and sleep quality for dependent groups. In order to reduce the relevant *β*-error, a high level of significance was chosen (*α* = .20). The results confirmed the likeness of the experimental groups for each of the demographic variables (*P* > .20).

The measurements and the experimental procedures were in compliance with American Psychological Association (APA) ethical principles. We considered a written informed consent from all participants about the measurements and the experimental procedures as sufficient, as the study only induced negligible risks; that is, it did not involve any foreseeable risk, harm, or discomfort. This procedure of not involving a research ethics committee approval is in line with APA regulations.

### 2.2. Procedure

After identifying lunch breaks as important recovery occasions in a presurvey, we invited interested parties to an informative meeting through internal communication by the management. In this meeting, the call center agents were asked to participate in an experimental study. All participants were screened in a short personal interview in order to ensure that they corresponded to our criteria of selection. Participants were told that the purpose of the study was to explore general effects on different ways to spend lunch breaks. However, no specific hypotheses were disclosed. In return for participation, participants were promised reports about both individual sleepiness profiles as well as the overall study findings. We did not compensate participating employees for their services. In an experimental field control group set-up lasting seven months (one month preintervention and six months during intervention from September to April), call center agents were randomly allocated to the experimental lunch break conditions: (a) 20 minutes of PMR or (b) 20 minutes ST break. The work task was the same for all participants and remained unchanged over the period of the study. For both groups, lunch break was scheduled between 12:00 and 13:00. Snacks were consumed by all participants during the first part of the lunch break (12:00–12:30). The second part of the break (12:30–13:00) took place (for the PMR group) in noise-subdued, dim-lighted (10 lux), opaque lockable cabins, called “silent rooms.” PMR instructions were given via wireless headphones (including calm instrumental background music) while participants lay on medical daybeds. The ST break was located in the company's staff room, where both participants and nonparticipants were involved in informal conversations, following their usual choice of small talk topics. Informal questioning revealed that this procedure was experienced as a regular, nonartificial way of spending lunch breaks, leading to no additional frustration or boredom in comparison to the prestudy lunch breaks.

Participants were instructed to maintain their regular behavior during the seven-month measurement period. Additionally, the PMR group was instructed not to practice PMR in their free time. All participants were questioned about their leisure time activities. None of the participants had to be excluded due to noncompliant behavior. Moreover, prior to each lunch break all subjects reported that they had abstained from alcohol, nicotine, and strenuous exercise for the past hour (included in self-report questionnaires). Caffeine consumption was determined at each self-report measurement both in terms of quantity and time of consumption; the self-report measurement took place five times per day once per month. There were no differences in daily consumption between the two groups (*P* < .05).

### 2.3. Measures

#### 2.3.1. Self-Report Scales

A well-proven, standardized, self-report sleepiness measurement, the German version of the Karolinska Sleepiness Scale (KSS) [29], served as primary outcome and was used to determine the sleepiness states. Participants had to choose the most appropriate description of their sleepiness level. On this one-item scale, ranging from 1 to 9, increased scores indicate increased sleepiness. In summary, we computed one KSS score per hour (average of 4 separate KSS ratings each 15 minutes), five times per day (*t*_KSS1_ = 12:00 h, *t*_KSS2_ = 13:00 h, *t*_KSS3_ = 14:00 h, *t*_KSS4_ = 15:00 h, and *t*_KSS5_ = 16:00 h), on seven working days (*d*_KSS1_–*d*_KSS7_) over the seven-month measuring period (*d*_KSS1_ = −0.50 months, *d*_KSS2_ = +0.50 months, *d*_KSS3_ = +1.50 months, *d*_KSS4_ = +2.50 months, *d*_KSS5_ = +3.50 months, *d*_KSS6_ = +4.50 months, and *d*_KSS7_ = +5.50 months) to determine the duration of PMR effects on sleepiness. The premeasurement *d*_KSS0_ (ST break for both groups) served as baseline findings. Moreover, the Pittsburgh Sleep Quality Index (PSQI) consisting of 18 items was applied as well as a 10-item questionnaire that capture caffeine consumption, lunch break activities, and a 3-item sleep diary (bed time, awakening time, and sleep duration).

#### 2.3.2. Reaction Time Parameters

To provide an objective measure of the participants' sleepiness, reaction time (RT) measurements serving as primary outcomes were recorded by the call center's inherent performance measurement system. On each working day, call center-specific standard performance indicators were recorded, specifically, the beginning and end of calls and other in-bound task-related indicators. The highly standardized structure of an in-bound call center agent's work-task loop can be described as follows: ringing (e0), accepting call (e1), starting introductory phrase (e2), coping with the customer's complaints/ending the call and logging-out (e3), postprocessing of call and logging-in (e4), and waiting for the next call/ringing (e5 = e0). Accordingly, a part of the call center agent's regular working task is to react as quickly as possible to an incoming call. The system recorded each workflow event with time stamps.

The RT in this study was operationalized as a time difference between ringing (e0) and accepting a call (e1). The average duration of a single work-task loop (and thus the interstimulus interval) was approximately three minutes. The corresponding RT was logged during the whole working day and was compared to the sleepiness indicating psychomotor vigilance task approach (cf. PVT) [30]. After this, the commonly applied PVT metric was calculated from the RT to capture the slowest RT, 90th percentile of RT (RT90), which indicates a prolonged reaction to a sustained attention demanding stimulus. In summary, we computed one RT90-measure per hour, eight hours per day (*t*_RT1_ = 8:00–8:59 h, *t*_RT2_ = 9:00–9:59 h, *t*_RT3_ = 10:00–10:59 h, *t*_RT4_ = 11:00–11:59 h, *t*_RT5_ = 13:00–13:59 h, *t*_RT6_ = 14:00–14:59 h, *t*_RT7_ = 15:00–15:59 h, and *t*_RT8_ = 16:00–16:59 h), 147 working days long (*d*_1_–*d*_147_), on 14 call center agents resulting in 8 × 147 × 14 = 16,464 indicators over the seven-month measuring period. The missing data (due to technical problems, holidays, and illness; in total 2.9%) of a participant on a specific time of day was replaced by stochastic regression. That is to say, the corresponding time-of-day data of the remaining days were applied as predictors for the missing value [31].

#### 2.3.3. Manipulation Check

We checked compliance and quality of relaxation by (a) applying a Visual Analogue Scale (VAS) [32] to rate depth of PMR relaxation (ranging from 0 = “no relaxation” to 100 = “very deep relaxation”) and a checklist of relaxation symptoms during the PMR breaks, which involved questions about the feeling of heaviness in 16 different muscle groups. Furthermore, the monthly scheduled checks included (b) informal questioning of participants by nonparticipating colleagues about their activities during lunch breaks (e.g., how often they use the silent room for the PMR group or how often they participate in small talks for ST group) and subsequent completion of questionnaires designed to evaluate certain lunch break activities and (c) weekly masked observations by nonparticipating peer colleagues (two persons per group). Noncompliant behavior (nonadherence to the lunch break mode) could be extrapolated from the above-mentioned questionings and observations. Furthermore, reporting less than 50 percent of the relaxation symptoms served as exclusion criterion. None of the participants fell below this criterion. Moreover, the results of the informal questioning showed an average number of adequate PMR breaks during the 6-month experimental period of 3.6 per week (72%; SD = 0.2) and for the ST group 4.5 per week (90%; SD = 0.3). Major reasons for not conducting an ST and PMR break were private obligations, social obligations (solving within-group conflicts and emotional support for colleagues), and falling asleep while practicing PMR (less than 20% of the whole PMR break trials).

### 2.4. Statistical Analysis

We conducted a priori power analysis for interaction effects of repeated measure ANOVA to determine the statistical power. Based on an estimated medium effect size of *f* = 0.3, a type I error of *α* = .05, a number of groups of *l* = 2, a number of repeated measurement of *k* = 7, and a sample size of *n* = 7 subjects per cell (total data points of self-report measures: 5 × 7 × 7 = 245 and objective measures: 8 × 147 × 7 × 2 = 16,464), it was computed that for both the self-report data and the objective data the power exceeds the 80% power for the significance tests of interaction effects.

For the subjective (objective, resp.) measures three (“immediate, intermediate, and anticipatory” × “RT90”; 3 × 1 = 3) two-dimensional repeated ANOVAs (Treatment × Measurement Day) were used to examine the main effects (factor Treatment) of postday (*d*_KSS2_–*d*_KSS7_; resp., *d*_RT22_–*d*_RT147_) differences between ST and PMR groups. Partial eta-squared as effect-size measure was used to examine sleepiness changes on each Time (*t*_KSS1_–*t*_KSS6_) separately. Post hoc comparisons were used to clarify differences of PMR and ST within *d*_KSS0_ to *d*_KSS6_ separately. In order to assess the change of the PMR effects, we calculated linear regressions and their b-weights over each immediate, intermediate, or anticipatory effect, calculated daily within the total 147 days of the intervention period. The onset and offset are determined by the monthly averaged values of PMR and ST, on which separate *t*-test was applied for each month.

Besides, the pooled postday (*d*_KSS2_–*d*_KSS7_; resp., *d*_RT22_–*d*_RT147_) perspective using two-way repeated measure mixed ANOVAs (Treatment × Time) summarizes the average postday time course. Greenhouse-Geisser corrections appropriate to dependent repeated measures were used, when the sphericity assumption was violated. Similar procedure *t*-tests were used to evaluate differences between PMR and ST groups. Following the reasoning of Perneger [33], who provides the most convincing arguments despite some controversy on the subject [34], no alpha level corrections were made for multiple testing. Partial eta-squared was computed as effect size measure. The statistical analyses were conducted using SPSS for Windows release 17 (SPSS, Inc., Chicago, IL, USA).

## 3. Results

### 3.1. Sample Characteristics and Preliminary Analyses

**Figure 1:**
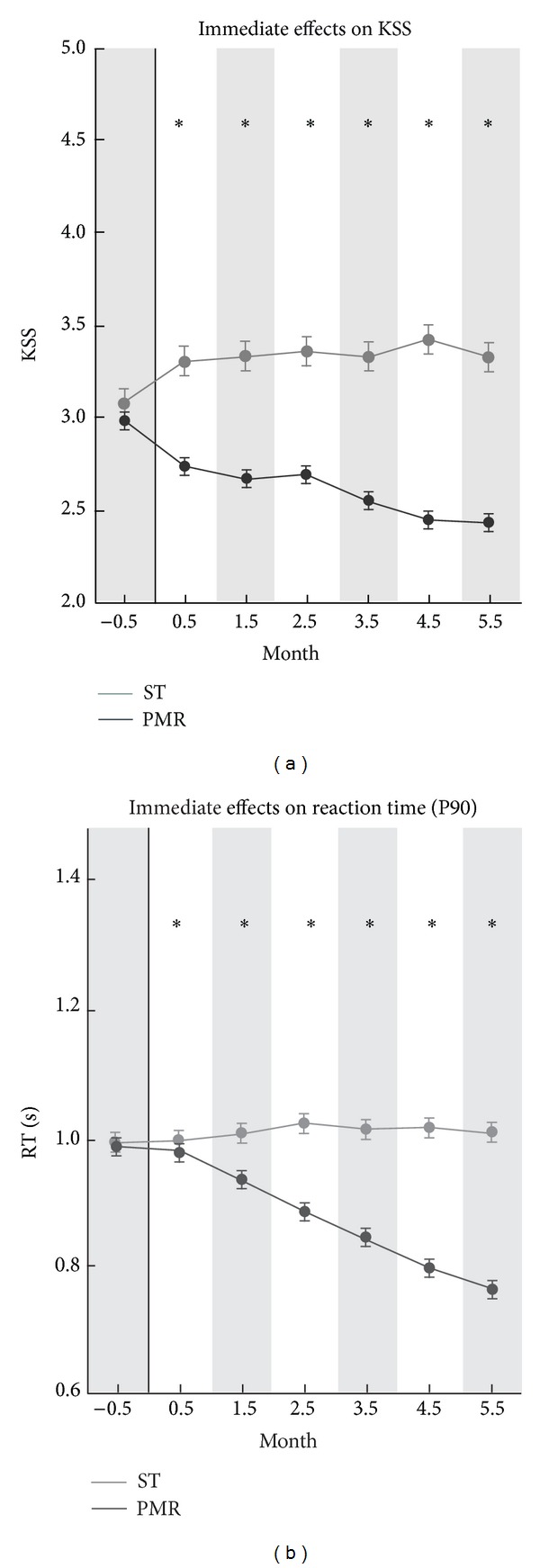
Immediate (postlunch break) effects on KSS and RT90 sleepiness KSS (a); RT90 (b) for the total preexperimental (−0.5 month, *d*_1_ to *d*_21_) and experimental period (−0.5 month to +5.5 months, *d*_22_ to *d*_147_) for PMR and ST group. Data are shown as mean ± SEM. ST group: grey color; PMR group: black color; ∗: significance of post hoc comparison (*P* < .05).

**Figure 2:**
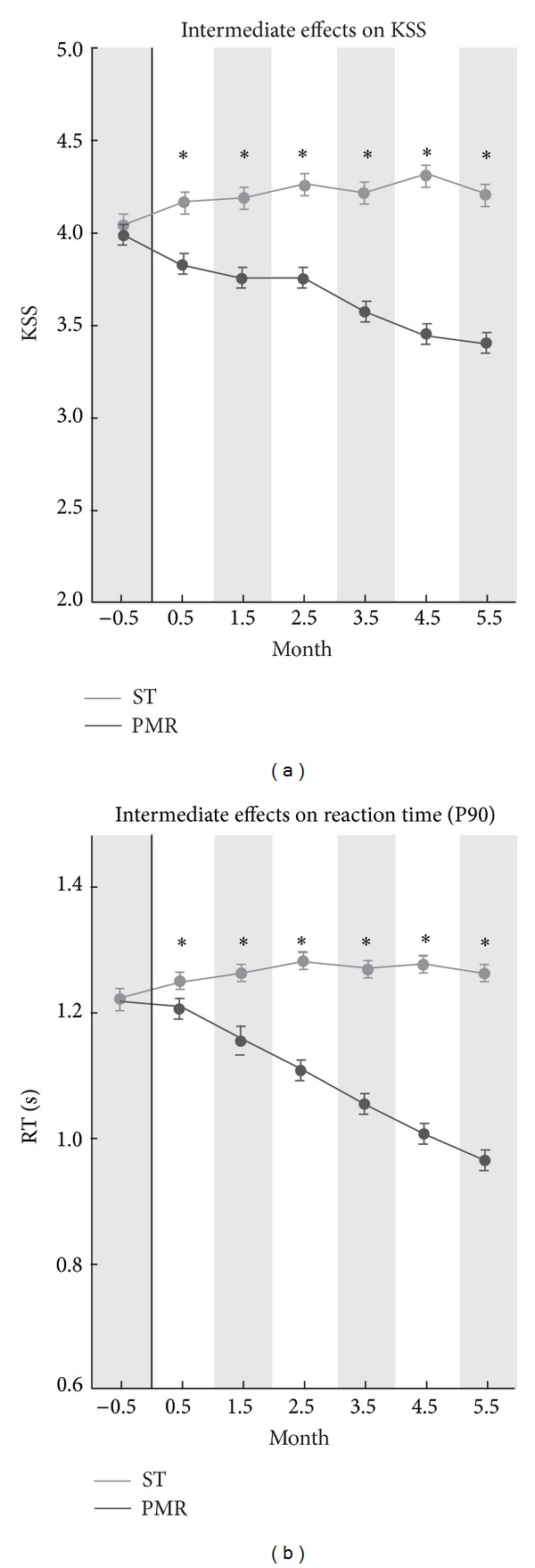
Intermediate (afternoon) effects on KSS and RT90 sleepiness KSS (a); RT90 (b) for the total preexperimental (−0.5 months, *d*_1_ to *d*_21_) and experimental period (−0.5 month to +5.5 month, *d*_22_ to *d*_147_) for PMR and ST group. Data are shown as mean ± SEM. ST group: grey color; PMR group: black color; ∗: significance of post hoc comparison (*P* < .05).

**Figure 3:**
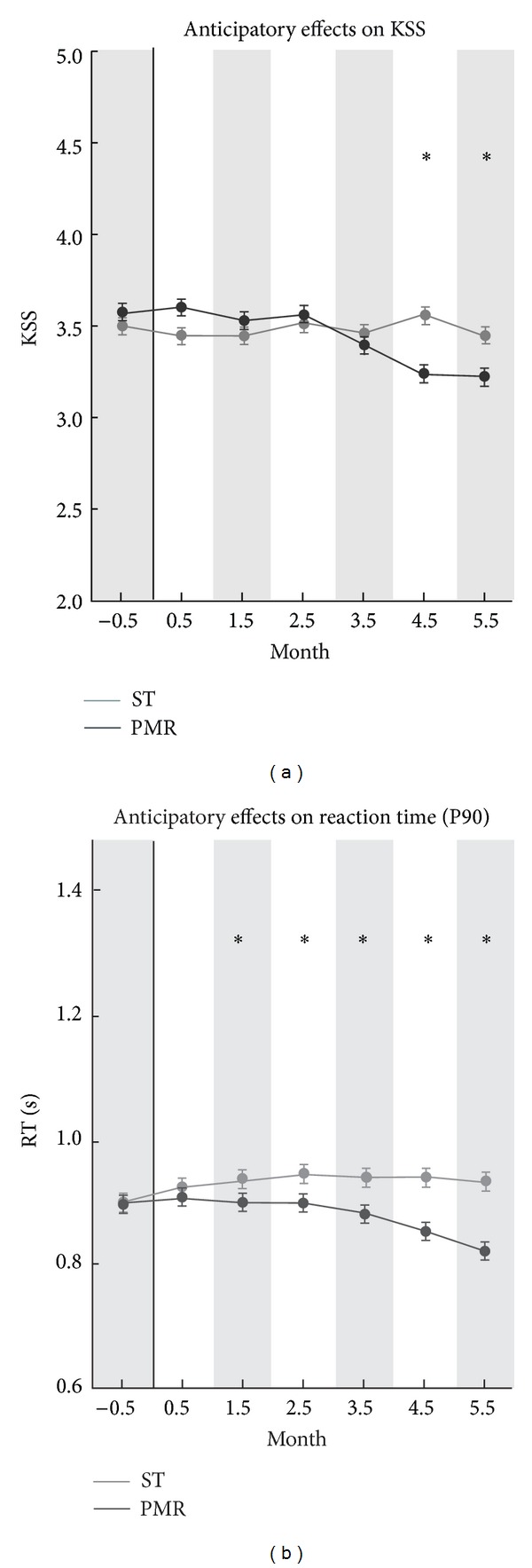
Anticipatory (morning) effects on KSS and RT90 sleepiness KSS (a); RT90 (b) for the total preexperimental (−0.5 months, *d*_1_ to *d*_21_) and experimental period (−0.5 month to +5.5 month, *d*_22_ to *d*_147_) for PMR and ST group. Data are shown as mean ± SEM. ST group: grey color; PMR group: black color; ∗: significance of post hoc comparison (*P* < .05).

Table 1 shows the main characteristics of the sample separately for the PMR and ST groups. The groups were statistically indistinguishable with regard to several sleepiness-influencing variables (age, gender, sleep duration, and sleep quality) and fell into the typical range of sleep quality as determined in norm studies [35]. The absolute mean sleepiness of the premeasurement days showed no differences between PMR and ST: immediate effects *F*_KSS_(1,12) = 0.63, n.s., *F*_RT90_(1, 42) = 0.11, n.s.; intermediate effects *F*_KSS_(1,22) = 0.54, n.s., *F*_RT90_(1,42) = 0.77, n.s.; and anticipatory effects *F*_KSS_(1,22) = 0.35, n.s., *F*_RT90_ = 0.53, n.s. (see, resp., Figures 1, 2, and 3). These findings support the interpretation of treatment group differences as being caused by the experimental factor Treatment. Immediate, intermediate, and anticipatory effects are described separately in the next sections.

### 3.2. Immediate Effects (Postlunch Break Sleepiness)

The postlunch break sleepiness (13:00–13:59) revealed significant different time courses (with months) for, respectively, the PMR and ST groups as depicted in Figure 1. The average change of sleepiness indicators, which are aggregated over all postdays, showed in comparison to the sleepiness within the same group in the preexperimental phase −7.9% for PMR_KSS_ (−6.9% for PMR_RT90_) and +3.9% for ST_KSS_ (+4.2% for ST_RT90_). Accordingly, the results obtained from two ANOVAs, *F*_KSS_(1,82) = 253.05, *η* ^2^ = .76, *P* < .001, *F*_RT90_(1,248) = 4102.20, *η* ^2^ = .94, *P* < .001, replicated the lower postlunch break sleepiness in the PMR conditions in both sleepiness indicators. The general change of the immediate effects was estimated by a linear regression function calculated over each single daily immediate effect within the total 147 days of the intervention period; it showed a significant decrease of sleepiness (*b*_KSS_ = 0.0118, n.s. and *b*_RT90_ = 0.0004, n.s.). Moreover, monthly averaged values of PMR and ST showed (see Figure 1) the onset of immediate effects for both KSS and RT90 in the first month. These differences between PMR and ST remained significant till at least the sixth month after implementation.

### 3.3. Intermediate-Term Effects (Afternoon Sleepiness)

The afternoon sleepiness (14:00–15:59) yielded significantly different time courses over the months for the PMR and ST groups as depicted in Figure 2. The average change of sleepiness indicators, which are aggregated over all postdays, showed in comparison to the sleepiness within the same group in the preexperimental phase −8.0% for PMR_KSS_ (−7.1% for PMR_RT90_) and +3.8% for ST_KSS_ (+4.1% for ST_RT90_). Additionally, the results revealed from ANOVA main effects replicated these different courses of afternoon sleepiness for both the PMR and the ST group, *F*_KSS_(1,82) = 274.67, *η* ^2^ = .62, *P* < .001 and *F*_RT90_(1, 248) = 1458.70, *η* ^2^ = .85, *P* < .001. In general, during the 147 days of the intervention period, a significant decrease of sleepiness was found (*b*_KSS_ = −0.0927, *P* < .05 and *b*_RT90_ = −0.0015, *P* < .05) for PMR but not for ST condition (*b*_KSS_ = 0.0140, n.s. and *b*_RT90_ = 0.0001, n.s.). Moreover, monthly averaged values of PMR and ST showed (see Figure 2) the onset of intermediate effects for KSS in the first month and for RT90 in the second month. The differences between PMR and ST remained significant during the six months.

### 3.4. Anticipatory Effects (Next Day Prelunchtime Sleepiness)

The anticipatory effects (08:00–11:59) revealed significantly different time courses for the PMR and ST groups as depicted in Figure 3. The average change of sleepiness indicators, which are aggregated over all postdays, yielded in comparison to the sleepiness within the same group in the preexperimental phase −9.2% for PMR_KSS_ (−6.9% for PMR_RT90_) and +4.1% for ST_KSS_ (+4.2% for ST_RT90_). Analogically, the results obtained from the ANOVAs, *F*_KSS_(1,84) = 60.69, *η* ^2^ = .27, *P* < .001 and *F*_RT90_(1, 248) = 89.01, *η* ^2^ = .26, *P* < .001, revealed diminished morning sleepiness of the PMR group in both sleepiness indicators. The general change of the anticipatory effects was estimated by a linear regression function calculated over each single daily anticipatory effect within a total of 147 days; it showed a significant decrease of sleepiness (*b*_KSS_ = −0.0845, *P* < .05 and *b*_RT90_ = −0.0012, *P* < .05) for PMR but not for ST conditions (*b*_KSS_ = 0.0078, n.s. and *b*_RT90_ = −0.0001, n.s.). Moreover, monthly averaged values of both PMR and ST showed (see Figure 3) the onset of anticipatory effects for KSS in the fifth month and for RT90 in the second month. These differences between PMR and ST remained significant until the end of the intervention.

### 3.5. Daily Time Course of Sleepiness

**Figure 4:**
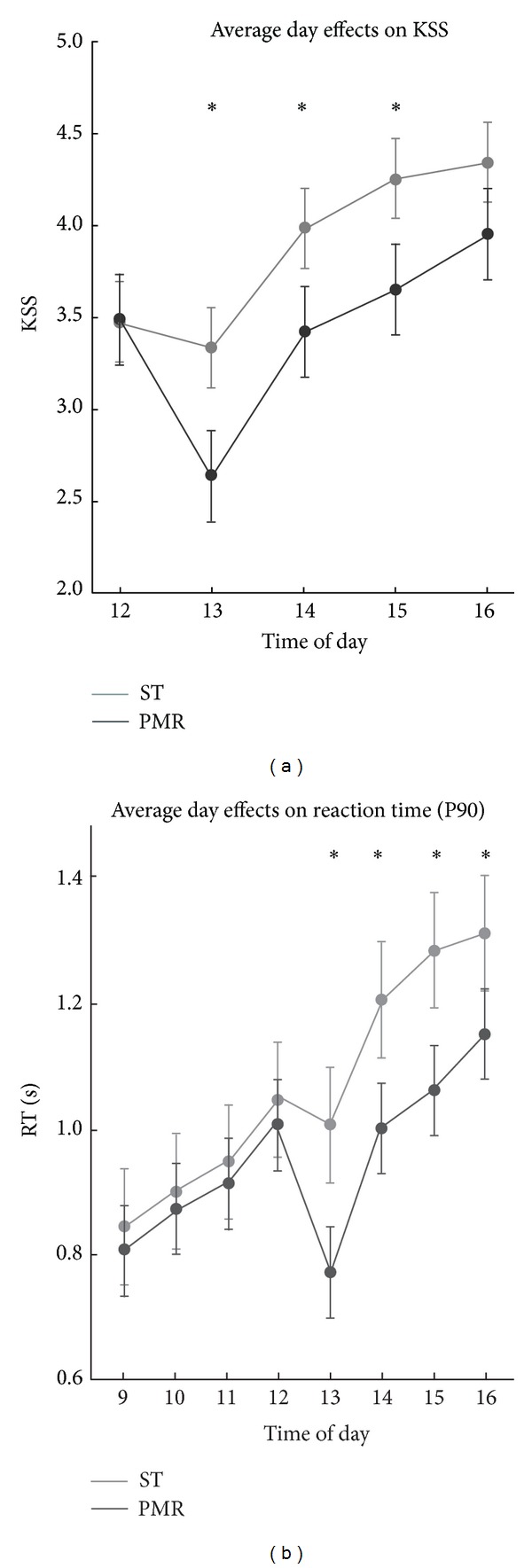
Time course of the KSS (a) and RT90 b) sleepiness states for an average postmeasurement day (−0.5 month to +5.5 months, *d*_22_ to *d*_147_; experimental period). Data are shown as mean ± SEM; ST group: grey color; PMR group: black color; ∗: significance of post hoc comparison (*P* < .05).

In order to summarize the daily time course of sleepiness for an average postday, we accumulated postdays (from the beginning of PMR implementation to the end; *d*_22_–*d*_147_; resp., *d*_KSS2_–*d*_KSS7_) to one average postday (see Figure 4). The average change of sleepiness indicators, which are aggregated over all postdays and time of day, yielded in comparison to the sleepiness within the same group in preexperimental phase −9.3% for PMR_KSS_ (−7.0% for PMR_RT90_) and +4.5% for ST_KSS_ (+4.2% for ST_RT90_). For each of the sleepiness measures, a 2-way ANOVA (2 Treatments × 8 Time intervals, respectively, five Time intervals), using the pooled sleepiness scores, revealed a significant interaction effect; it indicates distinct daily time courses for PMR and ST conditions *F*_KSS_(1,4) = 11.78, *η* ^2^ = .07, *P* < .001 and *F*_RT90_(1,7) = 424.90, *η* ^2^ = .79, *P* < .001.

## 4. Discussion

The aim of this worksite study is to evaluate the sleepiness influencing effects of salutogenic relaxation based lunch break within a fully realistic, long-term implementation into daily working life. Specifically, this seven-month experimental worksite field study addressed the question whether PMR lunch breaks reduce sleepiness using both subjective self-report measures and objective reaction time measures that were obtained from daily work tasks.

Immediate effects of relaxation can be observed across both sleepiness indicators. This corresponds to the strong immediate effect shown for relaxation in laboratory contexts [13, 36]. Except for the study by Krajewski et al. [17], to date, intermediate effects of several hours have not been included within existing PMR studies. Therefore, their appearance provides the first evidence for an extended range of PMR effects, which highlights the need to include postmeasurements of several hours after the PMR session to estimate the half-life time and the total impact of PMR recovery effects. In contrast, the commonly practiced ST break has shown an almost normal circadian rhythm as expected from previous literature [37]. Although much weaker anticipatory effects of relaxation were observed, results were significant for the first time. It can be speculated that slow adjustments in effort might lead to a redistribution of effort and, thus, daily activity. These changes are guided by the prospect of a relaxing break, which indirectly results in higher effort and lower prebreak sleepiness. Important conclusions, which can be drawn from these results, are that even measurements prior to the intervention should be included into an estimation of the total impact of an intervention. In summary, several empirical results and theoretical frameworks could be suitable for explaining the sleepiness reduction effect of PMR within self-care procedures [38]. In practicular the reduction of symptoms related to physical and mental fatigue, as well as emotional, motivational, and cognitive strain, might explain why PMR could reduce a range of symptoms, which are part of a general sleepiness experience and could lead to sleep-like regeneration.

Which interday trajectory will the PMR effects take? This question will help to determine the time scale for study designs of future self-care occupational intervention programs. Furthermore, estimating the trend will enable us to estimate the long-term stability and, therefore, the total impact and practical relevance of PMR-based lunch breaks. The starting point for the majority of PMR effects is mostly in the first month, showing a constantly increasing effect size over six months. This result is of high relevance for designing future studies evaluating the impact of PMR and self-care procedures in general, because it indicates that at least one month of learning and familiarization is needed to build expertise and trust, both prerequisites of deep PMR recovery.

Results showed an earlier sleepiness reducing effect in subjective measures than in objective measures. Subjective measures might benefit earlier from PMR because they are only influenced by one agent. Effects in a single reaction time based objective measure shows less clear results due to their dependence on the agents' effort and cognitive processing speed. Nevertheless, due to multiple testing, the reaction time measures show comparable results to the subjective indicators of sleepiness.

This intervention study does not claim to identify single isolated determinants underlying the sleepiness reduction but rather focuses on ecological validity; this should be kept in mind when mentioning several limitations referring to internal validity. Specifically, the study builds evidence for the feasibility, acceptance, and efficacy of a PMR-based lunch break within totally realistic occupational daily life settings. Methodological difficulties referring to the frequency and quality of experimental PMR and ST break realization, therefore, may have occurred. Participants' compliance with the ST break can be considered high. It is supported by the fact that ST breaks serve as the most common and natural form of lunch breaks (as determined in the presurvey). Participants' compliance in the PMR break condition was confirmed by random observations and informal questioning. Nevertheless, it may be a matter of debate whether the observed performance-enhancing effects resulted from placebo effects, from characteristics of the “silent room” (e.g., silence and darkness), from the amount of mental workload of the recovery activity, from short periods of napping during PMR, or from the PMR itself. Identifying these isolated determinants is an important second step of a research chain; however, it is a necessary first step to prove the overall effectiveness of an intervention. This approach is often used in intervention evaluation studies that aim to evaluate the overall efficacy of a program [39, 40].

It is conceivable that imitation of the PMR break by the ST group might have occurred in leisure time. However, informal interviews gave no hint of this, and even if this imitation had occurred, the real difference between evening sleepiness of ST and PMR would have been underestimated. Furthermore, there are uncertainties to the explanation of the anticipatory results: they might be induced by mediator effects of changed activity or sleep pattern at home [41] rather than directly influenced by PMR breaks.

The sample size is quite small, in comparison to large-scale cross-sectional correlation designs; however, it is within the typical range of experimental worksite field studies [14]. We have tried to compensate for a potential lack of robustness, internal validity, and significance of the results by applying repeated measurements. Future research might attempt to use further physiological [42, 43], behavioral [44, 45], or acoustically based sleepiness measures [46, 47]. Finally, it might be of further interest to measure individual performance and productivity indices, which might be considered as relevant endpoints from a perspective of organizational effectiveness.

The present study was carried out in a real but small worksite [48]. In order to judge population validity properly, it is evident that clarification concerning the ability to generalize the results is needed. Although the sample coverage in the particular call center was reasonable (around 50%), the small sample size limits the extent to which we can extrapolate from this call center context to other professional sectors. That is to say, it is not clear if PMR has a similar effect in different work settings (e.g., those that do not involve a lot of talking on the phone as a work task).

In summary, the results of this longitudinal pilot study indicate that a PMR-based lunch break, as a salutogenic self-care procedure, may significantly reduce sleepiness for several hours (immediate and intermediate effect) in realistic daily work settings. Additionally, the current study extends prior research by revealing anticipatory effects of sleepiness prior and with the prospect of a PMR break. Moreover, the onset of PMR effects due to familiarization and learning seems to appear 30 days after practice has started. Finally, the study provides evidence for the long-term acceptance and sustainability of the salutogenic recovery focused lunch break regime utilizing silent rooms as an implementation module enabling them to include PMR within daily lunch break routines.

## Figures and Tables

**Table 1:** Sample characteristics for PMR and ST group indicating the a priori equivalence of the groups (*n* = 14).

	PMR (*n* = 7)		ST (*n* = 7)		*P*
Age (years)	38.57	(9.34)	37.29	(9.20)	n.s.
BMI (kg/m^2^)	24.26	(1.71)	21.90	(2.10)	n.s.
Awakening time (h)	6.40	(0.24)	6.31	(0.29)	n.s.
Sleep duration (h)	8.17	(0.56)	7.95	(0.34)	n.s.
PSQI-sleep quality	4.12	(0.85)	4.29	(0.73)	n.s.
